# *PLOS Global Public Health*, charting a new path towards equity, diversity and inclusion in global health

**DOI:** 10.1371/journal.pgph.0000038

**Published:** 2021-10-13

**Authors:** Catherine Kyobutungi, Julia Robinson, Madhukar Pai

**Affiliations:** PLOS Global Public Health, San Francisco, CA, United States of America

As researchers, teachers, practitioners, health care workers, activists, and humans in this world, we are at a pivotal moment in the history of global public health. Over the past few decades, the field of global health—one which, in the wake of the Declaration of Alma Ata, held such promise to overturn systems of colonization, exploitation, discrimination and inequitable access to healthcare—has sadly replicated some of the same systems that have perpetuated the worst inequities. Power continues to be concentrated in the hands of a few elites in a few high-income countries, while low and middle-income countries are left facing the hardest health challenges with the fewest resources [1].

The ongoing struggle for vaccine equity [2,3] during the Covid-19 pandemic–in which rich countries have the luxury of imagining a post-pandemic life, offering booster shots, and even wasting vaccines, while poor countries grapple with newer, more dangerous variants and a lack of available vaccines—is the best illustration of the power asymmetry inherent in global public health [4].

The Covid-19 pandemic, the Black Lives Matter and Women in Global Health movements, as well as ongoing calls to decolonize global health have all created space for uncomfortable but important conversations that reveal serious asymmetries of power and privilege that permeate all aspects of the field [4], including funding [5] and authorship of research and scientific publications [6–8]. In particular, lack of diversity at all levels in global health journals [9,10], and lack of affordability for authors from low and middle-income countries [11,12] are key concerns that journal publishers must address, if they care to go beyond diversity and inclusion pledges.

Just as power asymmetries between high-income and low-income countries have existed and been brought to the fore in recent times, power asymmetries also exist within regions in the global South and even within countries. There is growing awareness of how global health perpetuates epistemic injustice [13] and tends to privilege scholarship from the Global North [14].

A new journal in global public health, therefore, must welcome and hold these uncomfortable conversations, using research, academic discourse, and advocacy to deliberately tip the balance of power in the direction of social justice, equity and diversity. With the publication of *PLOS Global Public Health*, a global Open Access forum for public health research, we aim to reach across disciplines and regional boundaries to address the biggest health challenges and inequities facing our society today.

The mission of *PLOS Global Public Health* is to address deeply entrenched inequities in global health and make impactful research visible and accessible to health professionals, policymakers, and local communities. We are committed to amplifying the voices of underrepresented and historically excluded communities and are deliberate and intentional about equity, diversity, and inclusion at all levels–editors, editorial boards, peer reviewers and authors—to broaden the range and diversity of perspectives we learn from and advance the health of all humankind.

Equity, diversity and inclusion are core to the journal’s mission—this will be intentional at every level of the journal, from its leadership (i.e. Editors-in-Chief), to Section Editors and the Editorial Board, to the authors and communities to which it serves. We are deliberately and actively recruiting a diverse pool of experts from all geographies and identities and make an intentional effort to ensure intersectional representation from historically underrepresented and excluded groups. We have already announced 43 section editors (from 23 countries) who are providing strategic guidance on the journal’s mission and scope. A majority of the section editors are women, and Black, Indigenous and people of colour (BIPOC), and half are colleagues based in the Global South. We are recruiting a much larger panel of academic editors and they are based in well over 50 countries.

We will amplify the work of BIPOC experts, especially people from the Global South, Indigenous scholars, and individuals working and living within their impacted communities. We realize that we need diverse formats to include a diverse range of voices and have already used our blog (https://speakingofmedicine.plos.org/) to quickly publish thoughtful and provocative pieces. We intend to explore and experiment with other formats to share information in a more inclusive way. We are also engaging students and youth in our journal [15], and will find ways to amplify their voices.

We will avoid elitism: In the spirit of *PLOS ONE*, we will not focus on novelty or impact factors, but rather on the rigor of the research and its contribution to the base of academic knowledge.

We will be accessible: *PLOS Global Public Health* will ensure immediate, gold open access to all content, including manuscripts and data. Article processing charges and fees will not be a barrier to publication (for details, please see https://plos.org/publish/fees/ and read about the PLOS Global Equity Model https://plos.org/resources/global-equity-model/). We are aware of the limitations of publishing only English language articles and plan to work towards language accessibility and global communication of research.

We will strive towards being more feminist, anti-racist, anti-patriarchal, anti-ableist, anti-elitist, and anti-classist in our work. We are explicitly addressing and combatting parachute or helicopter research through PLOS’ policy on inclusion in global research [16]. Lastly, we will publish research that address health inequities wherever they occur, not just in low-income countries.

With regards to scope, we will publish ethically and methodologically rigorous research that impacts public health, and particularly encourage submissions reporting research into health inequities and efforts to increase diversity and inclusion in public health. We consider submissions in areas including but not limited to global health delivery; infectious diseases; non-communicable diseases; race and health; mental health, laboratory medicine; maternal, newborn, and child health; nutrition; sexual and reproductive health and rights; Indigenous health; environmental and planetary health; evidence use and policy; global health governance; social and behavioral health; humanitarian aid and conflict/displacement; injuries, trauma and global surgery; global health financing and trade; gender and health; and global health security.

*PLOS Global Public Health* welcomes quantitative and qualitative primary research that contributes to the base of academic knowledge, including interdisciplinary research articles, clinical trials, replication studies, and negative or null results; systematic reviews whose methods ensure the comprehensive and unbiased sampling of existing literature; submissions describing methods, software, databases, or other tools that meet the journal’s criteria for utility, validation and availability that adheres to appropriate study design and reporting guidelines.

With this new journal, we are setting ambitious goals, and we expect to be held accountable. We acknowledge that we need to go beyond pledges [17]. We will make mistakes as we go along, but we will learn from them and do better. We look forward to building and engaging with the global public health community around the world, in charting a new path towards equity, diversity and inclusion in global health.

Editorial note: An earlier version of this editorial was published on the PLOS blog, *Speaking of Medicine and Health*, on 14^th^ June 2021
